# Deleted in malignant brain tumors 1 (DMBT1) elicits increased VEGF and decreased IL-6 production in type II lung epithelial cells

**DOI:** 10.1186/s12890-015-0027-x

**Published:** 2015-04-08

**Authors:** Hanna Müller, Christian Nagel, Christel Weiss, Jan Mollenhauer, Johannes Poeschl

**Affiliations:** Department of Pediatrics I, Neonatology, University Hospital Essen, University Duisburg-Essen, Hufelandstr. 55, 45147 Essen, Germany; Division of Neonatology, Department of Pediatrics, University of Heidelberg, Im Neuenheimer Feld 430, 69120 Heidelberg, Germany; Institute of Medical Statistics and Biomathematics, University Hospital Mannheim, Ludolf-Krehl-Straße 13-17D, 68167 Mannheim, Germany; Molecular Oncology and Lundbeckfonden Center of Excellence NanoCAN, Institute for Molecular Medicine, University of Southern Denmark, JB Winsloews Vej 25, 5000 Odense C, Denmark

**Keywords:** DMBT1, VEGF, IL-6, Innate immunity, Lung epithelial cells

## Abstract

**Background:**

Deleted in malignant brain tumors 1 **(**DMBT1) is an innate defence protein expressed in the lungs of preterm infants and adults. Recent studies showed that DMBT1 is important in angiogenesis and can bind to different growth factors including VEGF. We aimed at examining relationships between VEGF and IL-6 levels to DMBT1 expression in the lungs of preterm and term infants and in lung epithelial cells *in vitro*.

**Methods:**

We examined by ELISA VEGF levels in 120 tracheal aspirates of 57 preterm and term infants and tested for correlation with different perinatal factors as well as with DMBT1 levels. To examine the effect of DMBT1 on VEGF and IL-6 expression we compared type II lung epithelial A549 cells stably transfected with a *DMBT1* expression plasmid (DMBT1+ cells) to A549 cells stably transfected with an empty expression plasmid (DMBT1- cells). The concentrations of VEGF and IL-6 were determined via ELISA in the supernatant of the unstimulated cells and after stimulation with LPS, TNFα and Phorbol-12-myristate-13-acetate (PMA).

**Results:**

The VEGF levels in the tracheal aspirates of preterm and term infants were significantly correlated with DMBT1 levels (p = 0.0032), the postnatal age (p = 0.0073) and the presence of neonatal infection/sepsis (p = 0.0002). Unstimulated DMBT1+ A549 cells showed significantly higher VEGF expression (p = 0.0017) than DMBT1- cells. Significantly elevated VEGF levels were also confirmed for DMBT1+ cells after stimulation with TNFα (p = 0.0008), LPS (p = 0.0232) and PMA (p = 0.0025). The IL-6 levels were comparable in DMBT1+ versus DMBT1- cells without stimulation (p = 0.6028), but they were significantly reduced in DMBT1+ cells after stimulation with TNFα (p = 0.0003), LPS (p = 0.0088) and PMA (p = 0.0039).

**Conclusions:**

The data indicate that DMBT1 promotes VEGF and suppresses IL-6 production in alveolar tissues, which could point to DMBT1 having a possible role in the transition from inflammation to regeneration and being a potentially useful clinical marker.

## Background

DMBT1, a member of the scavenger receptor cysteine-rich (SRCR) protein family, is known as a protein with functions in innate immunity, inflammation and epithelial cell differentiation [1-3]. Especially tissues with functions in maintaining a barrier to the environment as, for example, the lung or the gut show DMBT1 expression, which is up-regulated upon inflammation [4-6]. Recently, DMBT1 has been reported to also exert functions in angiogenesis by influencing proliferation, migration and adhesion of endothelial cells. These functions could include binding of DMBT1 to different growth factors such as vascular endothelial growth factor (VEGF) and epidermal growth factor (EGF) [7].

The vascular endothelial growth factor (VEGF) represents a proangiogenic growth factor produced by cells such as pneumocytes, keratinocytes, macrophages, hepatocytes, and smooth muscle cells [8]. In various tissues, VEGF plays a role in normal and pathological angiogenesis, endothelial cell differentiation, maintenance and repair of existing vessels, embryonic development, microvascular permeability, and inflammatory processes.

VEGF is highly expressed in the lung and has multiple functions in the pulmonary tissue: It is necessary for lung development as well as for the structural maintenance during adult life [9-11]. Furthermore, VEGF affects the growth, proliferation and differentiation of type II pneumocytes [12,13]. Type II pneumocytes express the VEGF receptor VEGFR-2 [14], and VEGF up-regulates surfactant protein B and C [13]. Several studies described a role of VEGF in acute and chronic disease of the lung such as acute respiratory distress syndrome (ARDS), emphysema or pulmonary hypertension, pulmonary fibrosis, sarcoidosis, and after lung transplantation [9,14,15]. Beyond that, VEGF plays also a role in respiratory diseases of preterm infants, in the alveolarization of preterm lungs with respiratory distress syndrome characterized by edema and pulmonary inflammation, and in lungs with bronchopulmonary dysplasia [8,13,16,17]. Furthermore, VEGF is important for the pulmonary endothelial cells by functioning as a mitogen, survival and differentiation factor, promoting angiogenesis, and regulating the permeability of the endothelium [9].

Recent evidence that DMBT1 and VEGF are functionally linked in the regulation of endothelial cells and neoangiogenesis [7] prompted us to analyze as to whether similar relationships exist in the respiratory tract.

## Methods

### Patients and tracheal aspirates

To determine the VEGF concentrations we measured 120 tracheal aspirates from 57 included preterm and term infants (27 males, 30 females) treated at the Perinatal Center of the University of Heidelberg. These infants were part of the patients included in earlier studies of DMBT1 levels [18,19]. The newly determined VEGF levels were analyzed for correlation with DMBT1 levels from these earlier studies. The study was approved by the Ethics Committee of the University of Heidelberg, Germany, and the parents agreed by informed consent. The mean gestational age was 27.5 ± 4.2 weeks (postmenstrual age, range: 23–40 weeks), and the mean birth weight was 1128 ± 839 g (range: 370 – 3610 g).

The tracheal aspirates were obtained by applying 0.5-1.0 ml 0.9% sodium chloride solution into the endotracheal tubes and suctioning after three breaths of manual or mechanical ventilation. To adjust for different dilution factors associated with this procedure, we normalized the measured concentrations of VEGF or DMBT1 to the total protein content, which was detected with the bicinchoninic acid (BCA) protein assay (Pierce, Rockford, IL, USA).

Different factors were tested for correlation with VEGF levels in the tracheal aspirates, including parameters which vary within a patients’ observational period (postnatal age in days and relative DMBT1 level in the tracheal aspirates, treatment with indomethacin and the occurrence of neonatal infection/sepsis or the need of surgery at distinct days of life; Table 1) as well as the following parameters, which remain constant for each patient: gestational age (postmenstrual age in weeks), birth weight, bronchopulmonary dysplasia (yes or no; defined as additional oxygen required at 36 weeks of corrected gestational age with abnormal radiological findings of the lung), asphyxia (yes or no), intraventricular hemorrhage (all grades), cerebral hemorrhage (intraventricular, cerebellar; yes or no), maximal grade of respiratory distress syndrome (RDS; no RDS or grade 1–4 according to Giedion et al. [20]), survival (yes or no), antenatal corticosteroids for lung development (complete, yes or no), maternal infection defined by increased C-reactive protein (>5 mg/l) often accompanied by maternal leukocytosis, fever and/or histological chorioamnionitis (yes or no), premature rupture of the membranes (yes or no, defined as more than 24 hours duration), small for gestational age (below the 10^th^ percentile; yes or no), number of days with infection/sepsis (mean, range), and maximal grade of retinopathy of prematurity (RPM; no RPM, stage 1–5) (Table 2).

**Table 1:** **Correlation of non-constant parameters with the VEGF concentration in 120 tracheal aspirates of 57 infants**

**Parameter**	**p-value**
Postnatal age (days)	**0.0073**
DMBT1 concentration in tracheal aspirates	**0.0032**
Infection/sepsis	**0.0002**
Treatment with indomethacin	0.1669
Surgery	0.8733

**Table 2:** **Correlation of constant parameters with the VEGF concentration in tracheal aspirates of 57 infants**

**Parameter**		**p-value**
Gestational age (postmenstrual age in weeks, mean ± standard deviation)	27.5 ± 4.2	0.9230
Birth weight (g, mean ± standard deviation)	1128 ± 839	0.6718
Bronchopulmonary dysplasia (n)	34 (60%)	0.3076
Asphyxia (n)	7 (12%)	0.4642
Intraventricular hemorrhage (all grades)	16 (28%)	0.2607
Cerebral hemorrhage (intraventricular, cerebellar, n)	17 (30%)	0.2782
Respiratory distress syndrome (n)	50 (88%)	0.7636
Maximal grade of respiratory distress syndrome (RDS)		0.4284
No RDS (n)	7 (12%)	
Grade 1 (n)	6 (11%)	
Grade 2 (n)	18 (32%)	
Grade 3 (n)	20 (35%)	
Grade 4 (n)	6 (11%)	
Survival (n)	49 (86%)	0.8802
Antenatal corticosteroids for lung development (complete, n)	32 (56%)	0.9443
Maternal infection (n)	23 (40%)	0.4403
Premature rupture of the membranes (n)	8 (14%)	0.2180
Small for gestational age (n)	15 (26%)	0.4428
Number of days with infection (median, range)	11 (0–80)	0.7470
Retinopathy of prematurity (n)	34 (60%)	0.9268
Maximal grade of Retinopathy of prematurity (RPM)		0.6947
No RPM	23 (40%)	
Stage 1 (n)	4 (7%)	
Stage 2 (n)	16 (28%)	
Stage 3 (n)	14 (25%)	
Stage 4 (n)	0	
Stage 5 (n)	0	

### Enzyme-linked immunosorbent assay (ELISA)

We used an ELISA (Human VEGF Quantikine ELISA Kit DVE00, R&D Systems GmbH, Wiesbaden-Nordenstadt, Germany) to determine the relative VEGF-A (here referred to as VEGF) levels in the tracheal aspirates. The assay is usable for tracheal aspirates in according to the recommendations of the company and the minimal detectable VEGF concentration is less than 5 pg/ml. The VEGF levels were expressed in pg VEGF/μg total protein in order to adjust for differential dilution factors. The DMBT1 levels determined in two earlier studies were used for the comparison, where they were expressed as percentage of total protein content: (μg DMBT1/μg total protein) × 100 [18,19].

### Stimulation with TNFα, LPS and PMA

Human lung epithelial A549 cells, which were used in this study, have the characteristic morphology of type II lung epithelial cells, show typical lamellar bodies and form confluent monolayers [21]. The cells were stably transfected with an expression plasmid coding for the largest (8 kb) DMBT1 variant (DMBT1+ cells) under the control of a constitutive promoter or with an empty expression vector (DMBT1- cells) as described previously [22].

A549 cells were cultured with DMEM medium without phenol red (PAN-Biotech, Aidenbach, Germany) containing 10% fetal bovine serum, 1% L-glutamine and penicillin/streptomycin. Hygromycin B, used for the selection of cells with insertion of the plasmids, was added at a final concentration of 500 μg/ml to keep the cells under selection pressure. Cultivation was performed at 37°C and 5% CO2.

In experiments without stimulation 7.5 × 10^5^ cells were cultured with medium containing fetal bovine serum (FBS) until 90% confluence in a well of a 6-well plate was reached. The medium was removed and replaced with FBS-free medium for 48 hours. The supernatants of the confluent cell culture were then carefully removed, centrifuged at 1,500 rpm to remove remaining cells, and then frozen at −20°C until determination of the VEGF and IL-6 concentrations. The confluent cells on the cell culture plates were removed with trypsin from the wells and counted with a Neubauer chamber. Trypan blue staining was used to count the viable cells, enabling a calculation of the VEGF and IL-6 concentration referred to the number of viable cells.

In the case of stimulation the cells were incubated in FBS-free medium with LPS (10 μg/ml for 48 hours), TNFα (10 ng/ml for 48 hours) and Phorbol-12-myristate-13-acetate (PMA; 50 ng/ml for 48 hours). After stimulation the supernatant was harvested and analyzed for VEGF and IL-6 as described above.

### Determination of VEGF and IL-6 concentrations in supernatants of A549 cells by ELISA

The expression of VEGF-A and IL-6 in the supernatants of A549 cells was measured with an ELISA for human VEGF and for human IL-6 (Human VEGF Quantikine ELISA Kit DVE00 and Human IL-6 Quantikine ELISA Kit D6050, R&D Systems GmbH, Wiesbaden-Nordenstadt, Germany). The assays are both usable for cell culture supernatants in according to the recommendations of the company. The minimal detectable VEGF concentration is less than 5 pg/ml and the minimal detectable IL-6 concentration is less than 0.70 pg/ml. The VEGF and IL-6 concentrations were then calculated in pg/ml/100,000 viable cells.

### Western blot

Western blot analysis was performed to confirm DMBT1 expression in DMBT+ versus DMBT1- cells by using a polyacrylamide gel and non-reducing conditions. The polyclonal antibody anti-DMBT1p84 was used to detect DMBT1 as described previously [7]. An antibody against heavy chain non-muscle myosin (NMMHC, DPC Biermann, Bad Nauheim, Germany), a structure protein like β-actin, was employed as reference protein to allow comparison of the different samples. After incubation with the secondary antibody anti-rabbit-IgG conjugated to horseradish peroxidase the analyzed proteins were detected and visualized by ECL Western Blotting Substrate.

### Statistics

All statistical calculations have been done with SAS software, release 9.3 (SAS Institute Inc., Cary, NC, USA). For categorical parameters, absolute and relative frequencies are given. Quantitative parameters are presented as mean value and standard error of mean (SEM) together with minimum and maximum. In the case of outliers the median value has been calculated. In order to estimate correlations between quantitative parameters (i.e. postnatal age, DMBT1 concentrations, gestational age, birth weight) and the outcome variable “VEGF level”, regression analyses have been used. Variance analyses have been performed for testing the influence of qualitative parameters on the outcome variable. To create a formula calculating the VEGF content of an unknown tracheal aspirate we performed a multiple regression analysis. All these analyses have been performed with the SAS procedure PROC MIXED where patients’ ID was considered as a random variable and the parameters being tested as fixed variables. Furthermore, we compared DMBT1+ versus DMBT1- cells regarding VEGF and IL6- levels using the 2 sample t-tests or Mann–Whitney U-tests as appropriate. Test results with p-values smaller than 0.05 were regarded as statistically significant. In cases of multiple comparisons we performed a correction for multiple testing.

## Results

### VEGF concentrations in tracheal aspirates

**Figure 1:**
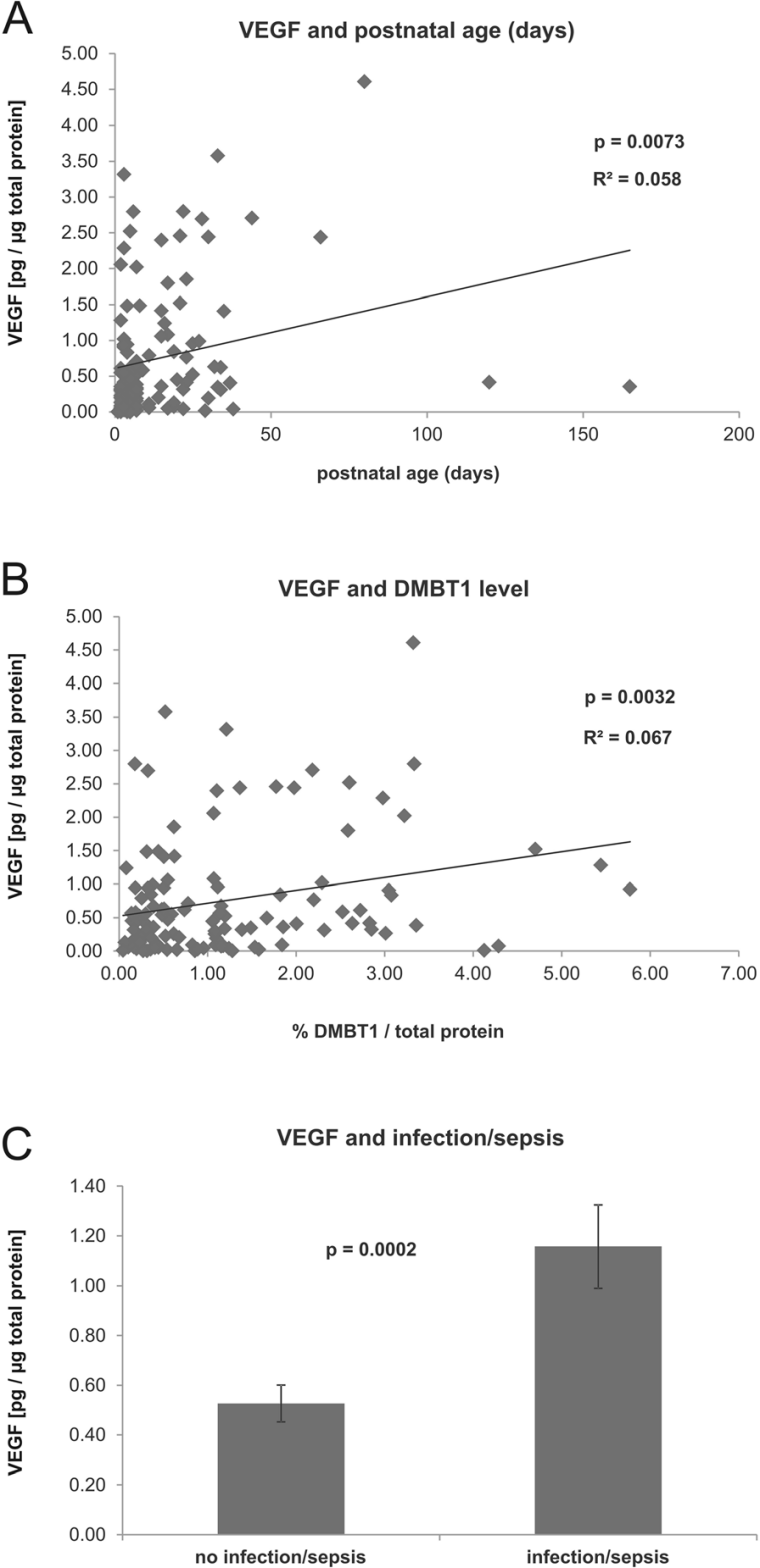
**Significant correlations between VEGF levels and tested parameters.** Correlations were established by testing of tracheal aspirates (n = 120) from infants and tested for significance via regression analysis or ANOVA using the SAS procedure PROC MIXED. **(A)** VEGF levels were significantly correlated with the postnatal age of the infants (p = 0.0073). **(B)** Positive correlation of VEGF levels with relative DMBT1 levels in the tracheal aspirates (p = 0.0032). **(C)** Significantly increased VEGF levels (mean ± SEM) in aspirates from infants with versus without infection/sepsis (p = 0.0002).

The mean VEGF-A (hereafter referred to as VEGF) concentration in the tracheal aspirates of the 57 preterm and term infants was 0.7524 ± 0.8882 pg/μg total protein with a range of 0.008 to 4.609 pg/μg total protein. The relative VEGF levels were significantly associated with the postnatal age, the earlier determined DMBT1 levels [18,19] in the tracheal aspirates and with neonatal infection/sepsis (Table 1, Figure 1). The other parameters, including gestational age, birth weight, bronchopulmonary dysplasia, respiratory distress syndrome and retinopathy of prematurity, showed no association with the relative VEGF levels of the tracheal aspirates (Table 2).

Furthermore, we performed a multiple regression analysis including these factors, which have been revealed as statistically significant, in the univariate analyses. This led to the following mathematical equation:

$$ y = 0.2840 + 0.007356{x}_1 + 0.1569{x}_2 + {x}_3 $$

where *Y* is the relative VEGF level (pg VEGF/μg total protein) in the unknown tracheal aspirate, which is significantly influenced by the parameters postnatal age (in days, *X*_1_, p = 0.0427), the relative DMBT1 level expressed as percentage of the total protein concentration (μg DMBT1/μg total protein × 100) in the tracheal aspirate (*X*_2_, p = 0.0159) and the presence or absence of neonatal infection/sepsis (*x*_3_ = 0.4891, if the infant has an infection/sepsis or *x*_3_ = 0 in the absence of infection/sepsis; p = 0.0038).

In conclusion, correlation of VEGF levels with postnatal age, infection/sepsis and DMBT1 levels is in good agreement with the findings that DMBT1 levels also correlated with postnatal age and infection/sepsis according to an earlier study [18].

### DMBT1 promotes basal and inducible VEGF expression in A549 cells

**Figure 2:**
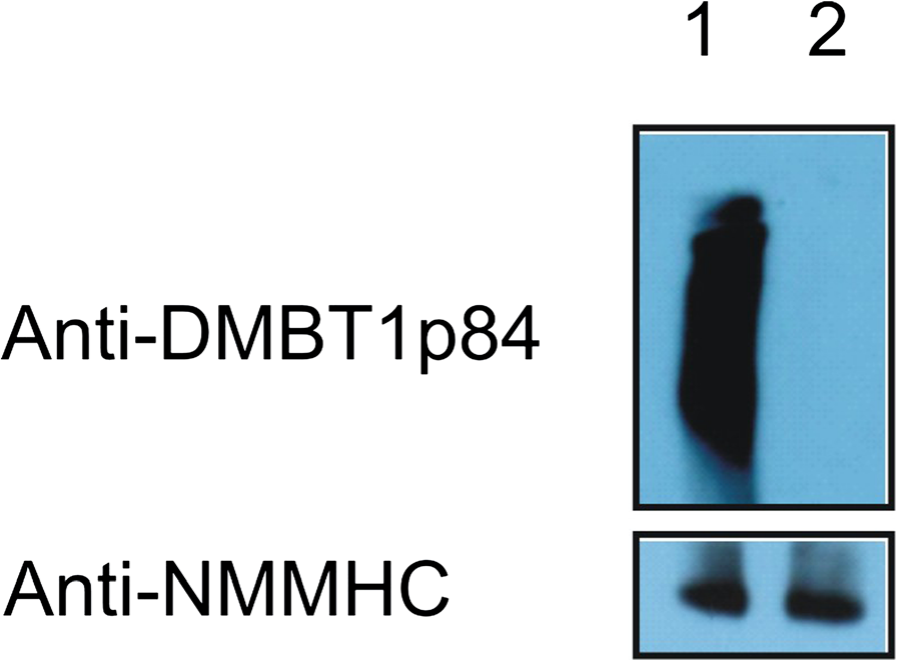
**DMBT1 expression in DMBT1+ and DMBT1- cells.** Western blotting using the polyclonal antibody anti-DMBT1p84 confirmed a high DMBT1 expression in DMBT1+ cells whereas no DMBT1 expression was observed in DMBT1- cells. Heavy chain non-muscle myosin (NMMHC), a structure protein like β-actin, was used as reference protein to examine equal loading of the samples. Lane 1: DMBT1+ cells, lane 2: DMBT1- cells.

Because the VEGF and the DMBT1 levels in the tracheal aspirates were significantly correlated, we next tested as to whether this might be based on a causal relationship. We therefore employed A549 lung epithelial cells with and without DMBT1 expression. DMBT1-positive A549 cells (referred to as DMBT1+ cells) were constructed using the expression plasmids described before [22]. Control cells were constructed in parallel using an empty expression plasmid. Western blot analyses confirmed expression in DMBT1+ cells, while no detectable protein levels were observed for A549 cells carrying the empty expression plasmid (Figure 2), so that we will refer to the latter as DMBT1- cells.

**Figure 3:**
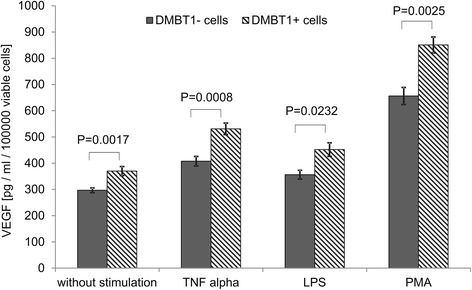
**VEGF levels in unstimulated and stimulated DMBT1+ and DMBT1- cells.** VEGF levels were determined in cell culture supernatants by ELISA. DMBT1+ cells showed significantly elevated VEGF levels in the basal state without stimulation as well as after stimulation with TNFα, LPS, or PMA. The VEGF mean values of the cell culture wells are shown for different stimulations and separately for DMBT1+ and DMBT1- cells. Error bars represent standard error of the mean. Sample sizes of the eight resulting subgroups were between 5 and 14. The p values were derived from 2 sample t-tests which have been used to compare the VEGF mean values for DMBT1+ and DMBT1- cells.

We next determined the basal VEGF levels in the two cell lines as well as the VEGF levels after stimulation with TNFα, LPS, or PMA. DMBT1+ cells displayed significantly elevated VEGF levels in the basal state as well as after stimulation, regardless of whether TNFα, LPS, or PMA was used (Figure 3). This clearly indicated that the presence of DMBT1 primes the lung epithelial cells for an enhanced VEGF production and confirms a causal relationship.

### DMBT1 suppresses IL-6 production in A549 cells

**Figure 4:**
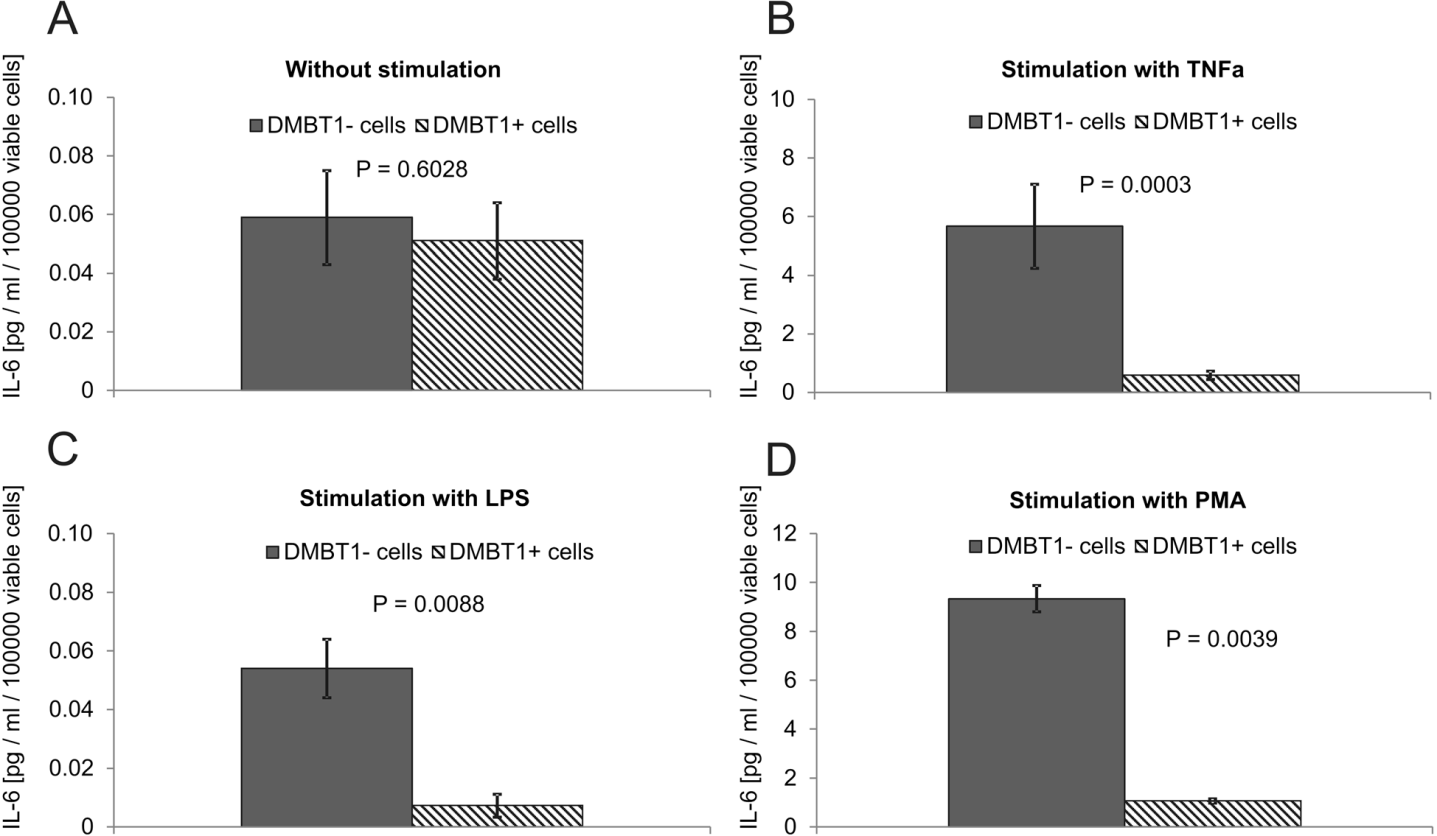
**Analysis of IL-6 levels in DMBT1+ and DMBT1- cells.** IL-6 levels were determined by ELISA in cell culture supernatants of DMBT1+ and DMBT1- cells **(A)** without stimulation, **(B-D)** after stimulation with the respective agents. In the absence of inflammatory stimuli, DMBT1 had no significant effect on IL-6 levels. Compared to DMBT1- cells DMBT1+ cells showed significant suppression of IL-6 production after stimulation with the pro-inflammatory stimuli (TNFα, LPS, and PMA). The means of 5–14 cell culture wells are shown. Error bars represent standard error of the mean. Statistical significance has been evaluated using Mann–Whitney U-tests because the data lacked normal distribution.

IL-6 is a well-known pro-inflammatory mediator in respiratory tract tissues, and, among various other effects, elicits increased VEGF production [23]. On the other hand, studies of intestinal inflammation in Dmbt1^−/−^ mice revealed that, in this tissue, DMBT1 may function in suppression of inflammation and IL-6 production via a role as pattern recognition molecule [5,6,24]. We thus evaluated next the IL-6 levels in alveolar epithelial A549 cells with and without DMBT1 expression. In the absence of inflammatory stimuli, DMBT1 did not elicit significant changes in IL-6 levels. By contrast, compared to DMBT1- cells, the DMBT1 expressing A549 cells displayed significant suppression of IL-6 production after stimulation with any of the three pro-inflammatory stimuli (TNFα, LPS, and PMA; Figure 4). This remarkably suggested that DMBT1 may substitute the IL-6 function in up-regulation of VEGF.

## Discussion

Proper spatio-temporal regulation of VEGF production is of critical importance for the respiratory tract. A distinct VEGF gradient is required for development of the airway tubular structures and lung endothelium, while VEGF overexpression may lead to dysmorphogenesis of the lung [9]. In pulmonary diseases like ARDS VEGF can aggravate the disease in a concentration-dependent manner by induction of endothelial permeability or can support the healing process. Different mechanisms are discussed to regulate intrapulmonary VEGF levels. Firstly, the loss of pneumocytes type II leads to decreased VEGF production. Secondly, proteases from inflammatory cells can degrade VEGF. Thirdly, more pulmonary VEGF can leave the lung across the alveolar-capillary barrier upon increased permeability. At fourth, soluble VEGFR-1, a splice variant of the membrane bound VEGFR-1, is a potent antagonist of the bioactive VEGF and influences the bioactivity of the VEGF present in the lung. Finally, other inhibitors of VEGF bioactivity in the lung contribute to its regulation such as ADAMTS1 and connective tissue growth factor [15,25]. Because understanding VEGF regulation is thus critical for understanding the basis of respiratory disease, we here addressed possible links between the secreted factor DMBT1 and VEGF.

### Influence of perinatal factors on VEGF levels in the tracheal aspirates of preterm and term infants

Our study revealed a correlation between the VEGF levels and postnatal age, but not with the gestational age or birth weight. This is in agreement with the study of Lassus et al., who examined 189 tracheal aspirates of 44 preterm infants in the first postnatal week and described an increase of VEGF concentration and no correlation with gestational age or birth weight [26]. However, in contrast to our study higher VEGF concentrations in infants of mothers with chorioamnionitis or premature rupture of the membranes and lower VEGF concentrations in infants with BPD or maternal preeclampsia were reported. Additionally, a negative correlation between VEGF levels and the number of surfactant doses was demonstrated in this study [26]. Another studies reported reduced VEGF levels in bronchopulmonary dysplasia of preterm infants, which is a neonatal chronic lung disease with reduced number of alveoles and reduced microvascular density [17,27]. Moreover, other authors reported that VEGF levels in tracheal aspirates of mechanically ventilated preterm neonates are not associated with BPD. Also in this study no correlation between birth weight and VEGF levels or gestational age and VEGF levels in tracheal aspirates could be detected [28]. In summary, our data confirm an increase of VEGF levels with postnatal age as reported by other authors [26].

### Correlation between pulmonary DMBT1 and VEGF levels in tracheal aspirates of preterm and term infants

The VEGF concentrations determined in this study for the tracheal aspirates of preterm and term infants showed a significant correlation with the DMBT1 levels determined earlier [18,19]. Confirmed causal relation was established by the significantly higher VEGF concentrations in the supernatants of the DMBT1+ cells in comparison to the DMBT1- cells. Thus, DMBT1 not only may interact with VEGF [7], but obviously is also a positive regulator of VEGF in type II lung cells.

### VEGF regulation in the respiratory tract

Inflammation and angiogenesis are intimately linked to each other. VEGF may affect immune surveillance and modulation [9]. IL-6 and TNFα, both involved in increased vascular permeability and remodeling, are able to induce VEGF expression and IL-6 as well as VEGF are up-regulated in response to hypoxia [25]. In early stages of acute lung injury the pro-inflammatory cytokines induce the expression and release of VEGF from pneumocytes type II, alveolar macrophages and neutrophils [25]. The observation that A549 cells constitutively produced VEGF was confirmed by different studies [29-31]. Clinical studies showed that LPS-induced lung injury is characterized by an increased pulmonary VEGF expression [25]. Prostacyclin-induced VEGF production is cyclooxygenase-2-dependent and PMA induced the expression of COX-2 [32,33]. PMA is an activator of the protein kinase C. The induction of VEGF by PMA via protein kinase C activation is also known from human endothelial cells [34]. Luo et al. reported about a non-significant increase of VEGF after stimulation of A549 cells with PMA (0.1 μM for 12 hours), but the stimulation with both, PMA and COX-2, led to a significant increase of VEGF expression [35]. In our study, we observed an increase of VEGF expression, when stimulating A549 cells with 50 ng/ml PMA for 48 hours. Other studies showed that inhibition of the protein kinase C led to significantly decreased VEGF secretion in primary human airway epithelial cells [36]. We observed that DMBT1 significantly elevated VEGF levels in type II lung cells in the basal state as well as after stimulation with pro-inflammatory agents. Within the light of the recently established role of DMBT1 in angiogenesis [7] it is thus conceivable that DMBT1, besides active participation in innate immunity, participates in the initiation of regeneration and/or in the transition from inflammation to regeneration in the respiratory tract.

### DMBT1-mediated IL-6 suppression

Pro-inflammatory stimuli induce a stimulation of NF-κB followed by induced transcription of pro-inflammatory mediators such as IL-6, COX-2 and TNFα. The pro-inflammatory cytokines IL-6 and TNFα are secreted to the tracheal aspirate fluid of infants with meconium aspiration syndrome [37]. Pulmonary IL-6 is increased in preterm infants developing BPD and in BPD lungs [38]. Further, the tracheal aspirate cells from preterm infants developing BPD showed an increased activation of NF-κB [39]. LPS (1 μg/ml for 24 hours) induced the expression of IL-1β, IL-6, IL-8 in A549 cells [40]. Under our experimental conditions, no increase of IL-6 after LPS-treatment was observed in the DMBT1- A549 cells and a decrease of IL-6 was found in the DMBT1+ cells. The up-regulation of IL-6 upon PMA stimulation [41] was confirmed in our study in the DMBT1- and DMBT1+ cells, but the effect is much higher in the DMBT1- cells (DMBT1- cells: 160–fold, DMBT1+ cells: 20-fold). The same is the case after stimulation with TNFα: The IL-6 increase is 100-fold in control cells and 10-fold in DMBT1 cells. Thus, we consistently observed that DMBT1 suppressed IL-6 in type II lung cells, when challenged with pro-inflammatory stimuli. This is in good agreement with data showing that Dmbt1^−/−^ mice were more susceptible to colitis and showed elevated mRNA levels for IL-6 and TNF during inflammation [5]. DMBT1 was up-regulated in intestinal epithelial cells after stimulation with LPS or TNFα, but is also able to inhibit LPS-induced NF-κB activation and cytokine secretion [6]. In intestinal epithelial cells DMBT1 is known to interact with LPS and to antagonize the actions of LPS [42]. Taken together, this may point to anti-inflammatory functions of DMBT1 in lung epithelial cells via suppression of IL-6 induction in the presence of pro-inflammatory stimuli, and that DMBT1 may substitute IL-6’s functions in up-regulating VEGF. This would be in good agreement with the assumption that DMBT1 may have additional functions at the transition from resolution of inflammation to regeneration in the respiratory tract and other tissues.

## Conclusions

Our data indicate that increased DMBT1 expression induces the production of VEGF and suppresses the IL-6 response in lung epithelial cells, which may point to a function in the transition from inflammation to regeneration.

## Abbreviations

DMBT1: Deleted in malignant brain tumors 1
VEGF: Vascular endothelial growth factor
PMA: Phorbol-12-myristate-13-acetate
RDS: Respiratory distress syndrome
BPD: Bronchopulmonary dysplasia
